# Supplementary material for: Proteomic and Metabolic Analysis of Pinus halepensis Mill. Embryonal Masses Induced under Heat Stress
Source: Int J Mol Sci. 2023 Apr 13;24(8):7211. doi: 10.3390/ijms24087211 (PMC10139065; doi:10.3390/ijms24087211)
Supplement: Supplementary file 1 [file ijms-24-07211-s001.zip › ijms-2274653-supplementary.pdf]

International Journal of
*Molecular Sciences*

MDPI

# Supplementary Material

**Table S1.** Proteins differentially accumulated in *Pinus halepensis* EMs induced under different temperatures treatments, according to the Kruskal-Wallis test ($p < 0.05$) and Dunn´s test of multiple comparisons (Benjamini-Hochberg $p$-value adjustment): Cond1 = 23 ℃; Cond2: 40 ℃ (4 h); Cond3: 50 ℃ (30 min); Cond4: 60 ℃ (5 min).

| | | | Dunn's Test of Multiple Comparisons | | | Kruskal-Wallis |
|---|---|---|---|---|---|---|
| **Protein** | **Uniprot Accession** | **Species** | **Cond2/ Cond1** | **Cond3/ Cond1** | **Cond4/ Cond1** | ***p* - value** |
| Citrate synthase. mitochondrial | O80433 | *Daucus carota* | 0.350 | 0.056 | 0.003 | 0.004 |
| Aconitate hydratase. cytoplasmic | P49608 | *Cucurbita maxima* | 0.017 | 0.002 | 0.081 | 0.006 |
| NADP-dependent malic enzyme 3 | Q9XGZ0 | *Arabidopsis thaliana* | 0.069 | 0.478 | 0.056 | 0.006 |
| Trifunctional UDP-glucose 4.6-dehydratase/UDP-4-keto-6-deoxy-D-glucose 3.5-epimerase/UDP-4-keto-L-rhamnose-reductase RHM1 | Q9SYM5 | *Arabidopsis thaliana* | 0.275 | 0.061 | 0.021 | 0.009 |
| Agglutinin | P06750 | *Ricinus communis* | 0.026 | 0.018 | 0.006 | 0.011 |
| Protein kinase G11A | Q0DCT8 | *Oryza sativa* subsp. *japonica* | 0.080 | 0.017 | 0.007 | 0.014 |
| Polyadenylate-binding protein RBP47 | Q9LEB3 | *Nicotiana plumbaginifoli a* | 0.009 | 0.015 | 0.056 | 0.015 |
| Molybdenum cofactor sulfurase | Q8LGM7 | *Solanum lycopersicum* | 0.116 | 0.010 | 0.014 | 0.016 |
| Actin-depolymerizing factor 10 | Q9LQ81 | *Arabidopsis thaliana* | 0.188 | 0.017 | 0.015 | 0.017 |
| 40S ribosomal protein S15a-1 | P42798 | *Arabidopsis thaliana* | 0.129 | 0.118 | 0.116 | 0.018 |
| 20 kDa chaperonin. chloroplastic | O65282 | *Arabidopsis thaliana* | 0.011 | 0.025 | 0.028 | 0.018 |
| NADPH--cytochrome P450 reductase 1 | Q9SB48 | *Arabidopsis thaliana* | 0.181 | 0.006 | 0.072 | 0.019 |
| Eukaryotic translation initiation factor 2 subunit beta | Q41969 | *Arabidopsis thaliana* | 0.016 | 0.015 | 0.144 | 0.019 |
| Histone H4 variant TH091 | P62786 | *Triticum aestivum* | 0.352 | 0.061 | 0.033 | 0.023 |
| Histone H2A.2.2 | P02277 | *Triticum aestivum* | 0.139 | 0.020 | 0.015 | 0.023 |
| Dynamin-related protein 12A | Q39821 | *Glycine max* | 0.139 | 0.015 | 0.020 | 0.023 |
| Probable fructokinase-7 | Q9FLH8 | *Arabidopsis thaliana* | 0.027 | 0.025 | 0.017 | 0.024 |

| | | | | | | |
|---|---|---|---|---|---|---|
| Pyruvate dehydrogenase E1 component subunit beta-4. chloroplastic | Q10G39 | *Oryza sativa* subsp. *japonica* | 0.403 | 0.048 | 0.033 | 0.025 |
| Chaperonin CPN60. mitochondrial | P35480 | *Brassica napus* | 0.007 | 0.130 | 0.097 | 0.025 |
| 6-phosphogluconate dehydrogenase. decarboxylating 1 | Q9LI00 | *Oryza sativa* subsp. *japonica* | 0.401 | 0.129 | 0.018 | 0.026 |
| 60S ribosomal protein L18-3 | Q940B0 | *Arabidopsis thaliana* | 0.380 | 0.049 | 0.069 | 0.028 |
| Chalcone synthase | P30079 | *Pinus sylvestris* | 0.075 | 0.162 | 0.012 | 0.031 |
| Diacylglycerol O-acyltransferase 1 | Q9SLD2 | *Arabidopsis thaliana* | 0.021 | 0.038 | 0.037 | 0.032 |
| Glutathione reductase. cytosolic | P48642 | *Oryza sativa* subsp. *japonica* | 0.071 | 0.043 | 0.455 | 0.032 |
| Pyruvate dehydrogenase E1 component subunit beta-1. mitochondrial | Q6Z1G7 | *Oryza sativa* subsp. *japonica* | 0.060 | 0.064 | 0.015 | 0.034 |
| Glutamyl-tRNA(Gln) amidotransferase subunit B. chloroplastic/mitochondrial | Q2R2Z0 | *Oryza sativa* subsp. japonica | 0.160 | 0.021 | 0.032 | 0.036 |
| 25.3 kDa vesicle transport protein | Q94AU2 | *Arabidopsis thaliana* | 0.056 | 0.024 | 0.029 | 0.036 |
| Ubiquitin carboxyl-terminal hydrolase 13 | Q84WU2 | *Arabidopsis thaliana* | 0.045 | 0.287 | 0.171 | 0.037 |
| Serine/threonine-protein phosphatase 2A 65 kDa regulatory subunit A gamma isoform | Q38951 | *Arabidopsis thaliana* | 0.093 | 0.162 | 0.204 | 0.038 |
| 3-isopropylmalate dehydrogenase. chloroplastic | P29696 | *Solanum tuberosum* | 0.385 | 0.061 | 0.049 | 0.038 |
| Alcohol dehydrogenase | P17648 | *Fragaria ananassa* | 0.321 | 0.097 | 0.056 | 0.041 |
| Carbamoyl-phosphate synthase large chain. chloroplastic | B9EXM2 | *Oryza sativa* subsp. *japonica* | 0.401 | 0.072 | 0.040 | 0.042 |
| Transcription factor TGA5 | Q39163 | *Arabidopsis thaliana* | 0.058 | 0.098 | 0.520 | 0.042 |
| 40S ribosomal protein S12 | Q9XHS0 | *Hordeum vulgare* | 0.031 | 0.049 | 0.162 | 0.049 |

**Table S2.** One-way analysis of variance for expression of different transcripts detected in *P. halepensis* embryonal masses induced under different temperature conditions (23 °C, 9 weeks; 40 °C, 4 h; 50 °C, 30 min; 60 °C, 5 min).

| **Kruskal-Wallis** | **d*f*** | **$X^2$ test** | ***p* Value** |
|---|---|---|---|
| *20 KDA CHAPERONIN (CPN20)* | 3 | 0.5245 | n.s.[1] |
| *CHAPERONIN CPN60 (CPN60)* | 3 | 0.0745 | n.s. |
| *CITRATE SYNTHASE (PDHB)* | 3 | 0.2167 | n.s. |
| *PYRUVATE DEHYDROGENASE E1 COMPONENT SUBUNIT BETA- 1* | 3 | 0.1841 | n.s. |
| *6-PHOSPHOGLUCONATE DEHYDROGENASE, DECARBOXYLATING 1 (G6PGH1)* | 3 | 0.2401 | n.s. |
| *PYRUVATE DEHYDROGENASE E1 COMPONENT SUBUNIT BETA-4* | 3 | 0.8395 | n.s. |
| *TRIFUNCTIONAL UDP-GLUCOSE A,6-DEHYDRATASE/UDP-4-KETO-6DEOXY-D-GLUCOSE 3,5-EPIMERASE/UDP-4-KETO-L-RHAMNOSE-REDUCTASE RHM1 (RHM1)* | 3 | 0.5921 | n.s. |
| *NADPH-CYTOCHROME P450 REDUCTASE 1 (ATR1)* | 3 | 0.5959 | n.s. |
| *PROBABLE FRUCTOKINASE-7* | 3 | 0.6958 | n.s. |

[1] not statistically significant.

**Table S3.** One-way analysis of variance for concentration of sugars (μmol $g^{-1}$ FW) detected in *P. halepensis* embryonal masses induced under different temperature conditions (23 °C, 9 weeks; 40 °C, 4 h; 50 °C, 30 min; 60 °C, 5 min).

| **Sugars** | | | |
|---|---|---|---|
| **ANOVA** | **d*f*** | **F value** | ***p* value** |
| Sucrose | 3 | 3.7320 | 0.0330 |
| Glucose | 3 | 1.1930 | n.s.[1] |
| Fructose | 3 | 1.8890 | n.s. |
| Mannitol | 3 | 0.9782 | n.s. |
| Sorbitol | 3 | 0.1167 | n.s. |

[1] not statistically significant.

**Table S4.** One-way analysis of variance for concentration of amino acids (µmol $g^{-1}$ FW) detected in *P. halepensis* embryonal masses induced under different temperature treatments (23 ℃, 9 weeks; 40 ℃, 4 h; 50 ℃, 30 min; 60 ℃, 5 min).

| Amino acids<br>ANOVA | d*f* | F value | *p* value |
|---|---|---|---|
| Aspartic acid | 3 | 0.4546 | n.s.[1] |
| Glutamic acid | 3 | 0.5485 | n.s. |
| Asparagine | 3 | 1.6030 | n.s. |
| Serine | 3 | 0.8004 | n.s. |
| Glutamine | 3 | 4.4920 | 0.0181 |
| Histidine | 3 | 0.5378 | n.s. |
| Glycine | 3 | 3.9470 | 0.0277 |
| Threonine | 3 | 0.8126 | n.s. |
| Arginine | 3 | 0.3369 | n.s. |
| Alanine | 3 | 1.8050 | n.s. |
| Tyrosine | 3 | 0.5632 | n.s. |
| Cysteine | 3 | 4.2780 | 0.0213 |
| Valine | 3 | 0.3568 | n.s. |
| Metionine | 3 | 0.5148 | n.s. |
| Tryptophan | 3 | 0.2859 | n.s. |
| Phenilalanine | 3 | 0.0708 | n.s. |
| Isoleucine | 3 | 0.2505 | n.s. |
| Leucine | 3 | 0.3126 | n.s. |
| Lysine | 3 | 0.5958 | n.s. |
| Proline | 3 | 0.4094 | n.s. |

[1] not statistically significant.
